# Recovery and Characterization of Spermatozoa in a Neotropical, Terrestrial, Direct-Developing Riparian Frog (*Craugastor evanesco*) through Hormonal Stimulation

**DOI:** 10.3390/ani13172689

**Published:** 2023-08-22

**Authors:** Yineska Otero, Natalie E. Calatayud, Igli D. Arcia, Denise Mariscal, Diego Samaniego, Dionel Rodríguez, Karina Rodríguez, Jorge Guerrel, Roberto Ibáñez, Gina Della Togna

**Affiliations:** 1 Smithsonian Tropical Research Institute, Balboa, Ancón, Apartado 0843-03092, Panama; yineskaotero1210@hotmail.com (Y.O.); arciaid@si.edu (I.D.A.); dmaiscal@gmail.com (D.M.); samaniego.33@hotmail.com (D.S.); dionel946@gmail.com (D.R.); k_r_sanchez@hotmail.com (K.R.); guerrelj@si.edu (J.G.); ibanezr@si.edu (R.I.); 2 Facultad de Medicina Veterinaria, Universidad de Panamá, Bella Vista, Apartado 3366, Panama; 3 San Diego Zoo Wildlife Alliance, Beckman Center for Conservation Research, 15600 San Pasqual Valley Road, Escondido, CA 92025, USA; ncalatayudcrump@sdzwa.org; 4 Facultad de Ciencias Naturales, Exactas y Tecnología, Escuela de Biología, Universidad de Panamá, Bella Vista, Apartado 3366, Panama; 5 The Amphibian Survival Alliance, Apartado 0830-00689, Panama

**Keywords:** amphibian, anura, sperm, terrestrial, assisted reproductive technologies, hormonal stimulation, motility, morphology, osmolality, pH

## Abstract

**Simple Summary:**

Recurrent successful breeding of the critically endangered Vanishing Rainfrog (*Craugastor evanesco*) in captivity has been challenging. Therefore, the objective of this study was to determine the effect of hormonal stimulation on the production and quality of *C. evanesco* spermatozoa, aiming to develop an efficient and safe sperm collection protocol as a tool to help reproduce this species in the near future. The Gonadotropin-Releasing Hormone agonist, at a concentration of 4 micrograms per gram of body weight, stimulated the production of high-quantity and quality spermatozoa. This study also described the morphology of *C. evanesco* spermatozoa for the first time. These results contributed new and valuable knowledge on the reproductive biology of *C. evanesco* and for the development of other assisted reproductive technologies that might be applied to this and other species’ conservation programs.

**Abstract:**

The Vanishing Rainfrog (*Craugastor evanesco*) is an endemic and critically endangered frog species of Panama. It is suspected that 90% of the population has disappeared from the wild. Frogs were collected from the wild and brought to a Captive Breeding Program; however, accomplishing regular reproductive events for this species has been difficult. The objective of this study was to determine the effect of hormonal stimulation on the production and quality of *C. evanesco* spermatozoa, aiming to develop an efficient and safe sperm collection protocol as a tool to help reproduce this endangered species. Mature males received intra-peritoneal injections with one of six hormone treatments, including des-Gly10, D-Ala6, Pro-NHEt9—GnRH-A, Amphiplex or hCG. Urine samples were collected at 10 different time points post-injection. Quality assessments included sperm concentration, percentage motility, percentage forward progressive motility (FPM), osmolality, pH and morphology analysis. Our results indicate that the optimal treatment for the collection of highly concentrated sperm samples of *C. evanesco* is 4 µg/gbw GnRH, followed by Amphiplex and 2 µg/gbw GnRH as sub-optimal treatments and finally, 6 µg/gbw GnRH and 5 and 10 IU/gbw hCG as non-optimal treatments. GnRH-A at 4 μg/gbw and Amphiplex stimulated the production of samples with the highest sperm concentrations and quality, despite Amphiplex producing lower percentages of intact acrosome and tail. In contrast, hCG concentrations were not reliable inducers of sperm production, consistently showing lower concentrations, higher percentages of sperm abnormalities and more acidic spermic urine than that induced by Amphiplex and GnRH-A. Morphological assessments revealed that *C. evanesco* spermatozoa have a filiform shape with a large acrosome on the anterior part of an elongated head, a small midpiece and a long tail with two filaments joined together by an undulating membrane.

## 1. Introduction

**Figure 1:**
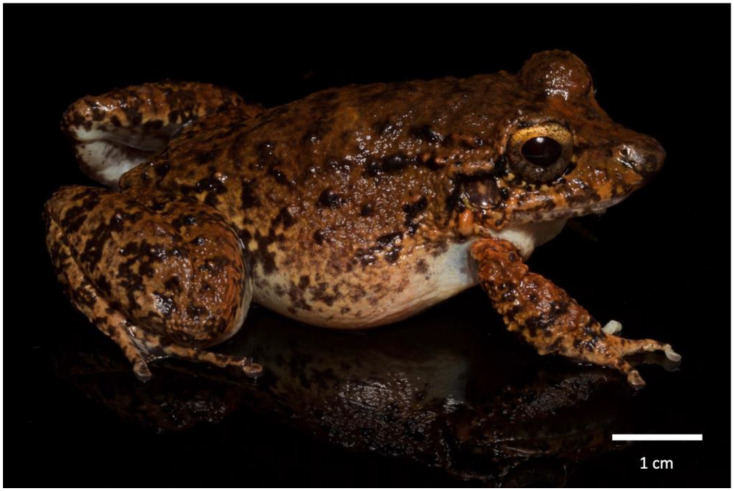
An adult male *Craugastor evanesco* from the Panama Amphibian Rescue and Conservation Project. Photo by Brian Gratwicke.

In response to the continued decline of its amphibian species, Panama has developed a Conservation Action Plan to help mitigate the severity of the problem [1]. An assessment conducted on the conservation status of Panamanian amphibians found that about one-third of them were under some degree of risk [2]. Amongst those described in the plan, *Agalychnis lemur* (CR), *Tripion spinosus* (NT-decreasing populations), *Pristimantis museosus* (VU), *Atelopus limosus* (CR), *Craugastor evanesco* (CR), *Strabomantis bufoniformis* (EN) and *Cruziohyla calcarifer* (LC-decreasing populations) were highlighted as conservation priorities for the country [1,3]. Of these, *Craugastor evanesco* (Figure 1) was the most recently described species (2010) and is endemic to Panama [4]. The species is highly susceptible to chytridiomycosis, a disease caused by the fungus *Batrachochytrium dendrobatidis* (*Bd*), and its common name, Vanishing Rainfrog, refers to the fact that it had mostly disappeared from its distribution range from the time it was discovered (2004) to its formal description (2010). It is estimated that 90% of its population has disappeared from the wild [3]. Species of the family Craugastoridae are terrestrial or riparian, lay fairly large eggs that are deposited terrestrially and undergo direct development [5].

In situ conservation programs for persisting amphibian species and populations in the wild are not enough to avoid ongoing declines and extinctions due to chytridiomycosis; thus, ex situ conservation programs have become the only hope for species like *C. evanesco* and are important tools to help mitigate this crisis [6]. Due to the urgency to save the species, forty sexually mature individuals (20 females and 20 males) were originally collected from the wild and brought to the Panama Amphibian Rescue and Conservation Project to establish an ex situ conservation program (ECP) for the species [7].

The primary goal of an ECPs is to maintain a self-sustainable captive population, that is reliably reproductive and which can retain at least 90% of the genetic variation present in the wild population for at least 100 years [8,9]. However, ex situ conditions usually alter the reproductive success of these species, and achieving regular reproductive events is a difficult task. Except for its description, there is no information available on the ecology, reproduction or natural history of the species; therefore, all current ex situ reproduction efforts for *C. evanesco* are based on scarce information on the natural histories of related species. To date, accomplishing recurrent reproductive events for this species has been challenging [7,10].

Assisted Reproductive Technologies (ARTs) are tools designed to help reproduce endangered species, and their development is usually species-specific [10,11,12]. Hormonal stimulation is an efficient technique that helps potentiate the reproductive success of ex situ populations. After adequate standardization, it can be used to collect high-quality gametes and manipulate the reproductive behavior and cycle of a species [10,12,13,14,15,16,17,18,19,20,21].

Previous studies by our laboratory have produced valuable information on the effect of hormonal stimulation on the production of spermic urine in endangered species from the genus *Atelopus* [10,12,22], resulting in safe and efficient sperm collection protocols for five of these species. These methods have later been applied to the collection of high-quality sperm for cryopreservation or synchronization of reproductive events [10]. Therefore, the objective of this study was to determine the effect of hormonal stimulation on the production and quality of *C. evanesco* spermic urine, aiming to develop an efficient and safe sperm collection protocol as a tool to help reproduce this critically endangered species in the near future.

## 2. Materials and Methods

### 2.1. Animals and Husbandry

From December 2018 to December 2019, thirty-one adult male *C. evanesco* (29.00 ± 7.32 g) housed at the Smithsonian Tropical Research Institute’s (STRI) Panama Amphibian Rescue and Conservation Project, located in Gamboa, Panama, were used for this study. Animal use protocols were submitted for the STRI’s Institutional Animal Care and Use Committee (IACUC) approval. Tanks (ExoTerra Terrarium, 24″ × 18″ × 24″), housing up to two frogs, containing rocks and substrate, were kept at 23–25 °C and misted with carbon-filtered water every 8 h for 3 min and drained through a bulkhead. UV lighting was set on a 12 h cycle with Zoomed 10.0 T8 high-output UV-B bulbs to avoid D3 hypovitaminosis and help with proper bone growth. Frogs were fed ad libitum three times per week with live crickets (*Acheta domestica*) and roaches (*Blaptica dubia*) dusted with a calcium carbonate powder containing vitamin D3 (Rep-Cal, New England Herpetoculture LLC, Plainville, CT, USA) or multivitamins with beta carotene powder (Repashy, New England Herpetoculture LLC, Plainville, CT, USA) on a rotating basis.

### 2.2. Hormone Stimulation and Sperm Collection

**Figure 2:**
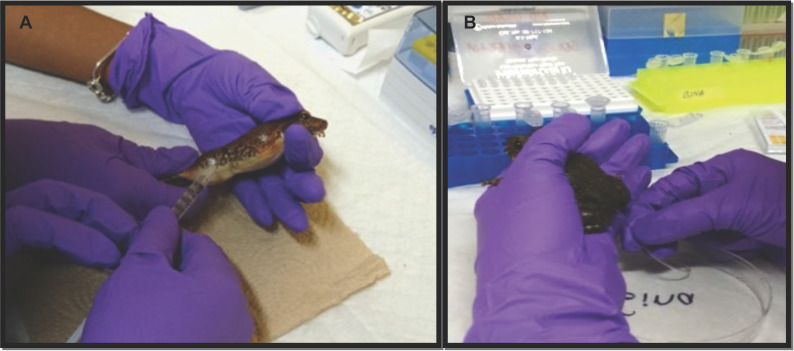
Hormonal stimulation and collection of *Craugastor evanesco* spermatozoa. (**A**) Intraperitoneal injection of hormonal treatments at a volume of 10 μL/gbw for each concentration. (**B**) Spermic urine collection by cloacal catheterization.

Males received intra-peritoneal (IP) injections (Figure 2A) with one of six hormone treatments including des-Gly^10^, D-Ala^6^, Pro-NHEt^9^—GnRH (GnRH-A; Sigma-Aldrich Corporation, St. Louis, MO, USA), Amphiplex—a cocktail of 0.4 μg GnRH-A plus10 μg/gbw metoclopramide hydrochloride, a dopamine antagonist (Sigma-Aldrich Corporation) or hCG (CG5 Chorionic gonadotropin human; Sigma-Aldrich Corporation) (Table 1). Two different controls were used: Control 1—males injected with Amphibian Ringer Solution (ARS) and Control 2—uninjected males. For each treatment, seven experimental males and four control males (two males for each control type) were used. Males used more than once were rested for a minimum period of four weeks between treatments. Before hormonal stimulation took place, urine was collected to ensure the absence of sperm. Treatments were chosen based on preliminary concentration–range studies on the species and previous successful spermiation inductions in other species (see review [10]).

Throughout spermic urine collection periods, animals were kept individually in temporary ventilated plastic containers (10″ × 6″ × 4″) lined with a dampened layer of paper towel (~15 mL of water) and covered from light to reduce stress. Spermic urine samples were collected at 10 different time points—0.5, 1.5, 2.5, 3.5, 4.5, 5.5, 6.5, 24, 28 and 48 h—post-injection by cloacal catheterization (Micro Medical Tubing—85 Durometer Vinyl, Scientific Commodities Inc., Lake Havasu City, AZ, USA) (Figure 2B). Samples were transferred to 1.5 mL tubes for evaluation.

### 2.3. Sperm Processing and Analysis

Quality assessments included sperm concentration, percentage motility, percentage forward progressive motility (FPM), osmolality and pH. Sample volumes were measured with a 200-μL pipette. Sperm concentration was assessed by adding 10 μL of the spermic urine sample to a hemocytometer. Osmolality and pH were measured using an Osmocheck urine analysis unit (Vitech Scientific, West Sussex, UK) and pH indicator strips (pH 5.0–10.0, EMD Millipore, Billerica, MA, USA), respectively. Percentage sperm motility was determined by counting all the cells with any type of movement from a total of 100 cells under a Zeiss AXIO Scope A1 microscope at 400× magnification. Percentage FPM was obtained by counting all the cells with forward progression relative to the 100 cells that were assessed for motility.

### 2.4. Sperm Morphological Assessment

In preparation for morphological assessments, 10 μL of spermic urine was fixed in 80 μL 4% paraformaldehyde (PFA) in phosphate-buffered solution (PBS). Fixed sperm cells were then stained with Coomassie blue (0.1% Coomassie Brilliant Blue R-250, 50% methanol and 10% glacial acetic acid) for 90 s, air dried and mounted with Permount™ Mounting Medium (Fisher Scientific, Pittsburgh, PA, USA) on Superfrost slides (Superfrost Plus ™, Thermo Scientific, Arendalsvägen, Göteborg, Sweden) [12]. Evaluations were conducted at 1000× magnification using a Zeiss AXIO Scope A1 microscope. Morphological assessments were carried out in two stages as described in Della Togna et al. [12]. Measurements were recorded by evaluating 100 cells for each feature using the ImageJ 1.5.3 software (National Institutes of Health, Bethesda, MD, USA). Normal morphology was categorized broadly by the presence of an intact acrosome, head and tail versus abnormal acrosome (missing or not intact), head (bent or broken) and tail (coiled, broken or with cytoplasmic droplet).

### 2.5. Sperm Concentration–Response Curves and Peaks

Sperm concentration–response curves describe the magnitude of the response in terms of sperm concentration as a function of exposure to different hormonal treatments and doses over time. This information denotes if and when a specific treatment’s effect begins, peaks and ends. Curves were calculated using descriptive statistics. For each collection time point, replicates were averaged and the standard deviation was calculated.

Identification of sperm concentration peaks allows researchers to identify the specific window of time in which collections of highly concentrated sperm samples should occur. When applying ARTs such as sperm cryopreservation and artificial fertilization, the use of such samples is required. Therefore, in this study, sperm concentration peaks are defined as the time points where the number of spermatozoa per unit volume of urine is higher compared to other time points. To obtain sperm concentration peaks for each treatment, cutoff values were used and calculated as follows: total mean sperm concentration for each time point was averaged and added to one times the standard deviation (SD). Time points with sperm concentrations equal to or higher than the cutoff were considered part of the peak and removed for the following round of calculations. The process was repeated with the remaining values until there were no time points equal to or higher than the cutoff [12,23]. Sperm concentration peaks were not statistically analyzed.

### 2.6. Statistical Analysis

Statistical analysis was conducted using GraphPad Prism 9.0 software (GraphPad Software, Inc., La Jolla, CA, USA). Statistical analysis for concentration, percentage motility, percentage forward motility, osmolality and pH were based on and applied to metrics obtained during the time points where peak concentrations occurred. If more than one peak was identified for a single treatment, all the metrics obtained during these peaks were included in the analysis. The effects of hormonal treatments on sperm concentration and percentage motility were determined using one-way ANOVA with Tukey’s post hoc test. The effects of hormonal treatments on percentage motility and FPM, osmolality, spermic urine pH and morphology parameters were determined with the Welch ANOVA test followed by Dunnett’s post hoc test (when variances were not homogeneous). The effect of hormonal treatments on sperm head and tail size was determined with two-way ANOVA followed by Šidák’s multiple comparison test. Values for all data were expressed as mean ± SD. Means were considered statistically different if *p* < 0.05.

## 3. Results

The results of this study indicate that all tested hormones (GnRH-A, hCG and Amphiplex), but not all concentrations, elicited the release of sperm in *C. evanesco*, with 100% of males responding to all concentrations of GnRH-A and Amphiplex, 44% to 10 IU/gbw hCG and no males responded after treatment with 5 IU/gbw hCG. GnRH-A at 4 μg/gbw and Amphiplex stimulated the production of samples with the highest sperm concentrations and quality, followed by GnRH-A at 2 and 6 μg/gbw. However, the hCG concentrations used in this study were not reliable inducers of sperm production and consistently had higher percentages of sperm abnormalities. Furthermore, spermic urine induced with hCG was more acidic than spermic urine induced by GnRH-A and Amphiplex.

### 3.1. Sperm Concentration Post Hormone Induction

**Figure 3:**
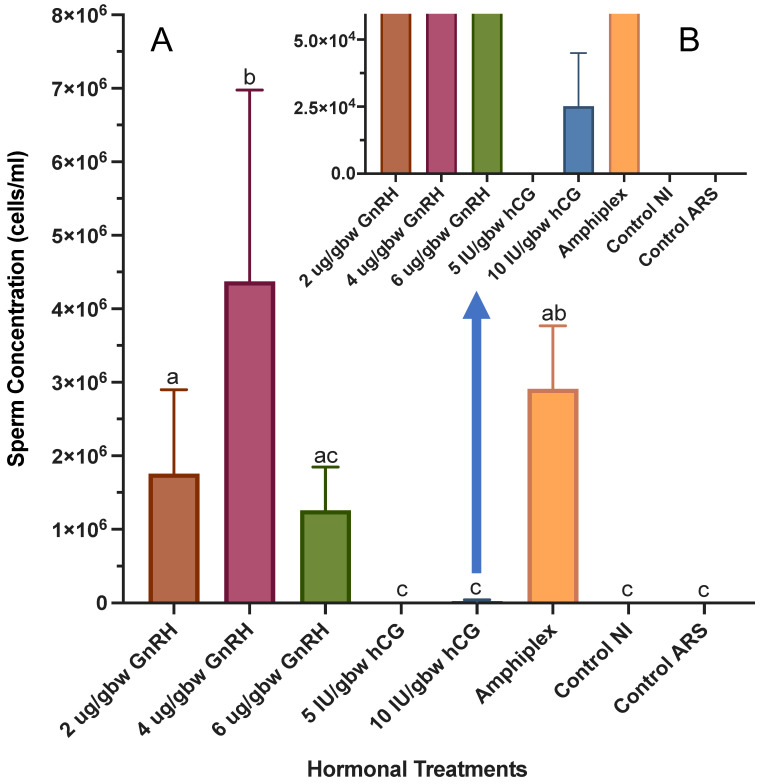
Concentration during sperm production peaks after hormonal stimulation. Six hormone treatments (*n* = 7 per treatment) and two control treatments (no hormone, *n* = 4 per treatment). (**A**) Comparison of sperm concentration among all treatments and controls. (**B**) Sperm concentration during sperm production peak obtained after treatment with 10 IU/gbw hCG at a lower scale for visualization. Significant differences in treatments are indicated by the differential lettering. Values for all data were expressed as mean ± SD. Means were considered statistically different if *p* < 0.05.

There was a significant effect of hormonal treatments on sperm production for *C. evanesco* (F_(7,51)_ = 17.31, *p <* 0.0001) (Figure 3).

Except for 5 IU/gbw hCG, all other treatments successfully produced a response resulting in a range of sperm concentrations from 2.78 × 10^3^ ± 5.33 × 10^3^ (10 IU/gbw hCG) to 8.64 × 10^6^ ± 2.00 × 10^7^ (4 μg/gbw GnRH-A). Control samples were devoid of sperm at all time points. GnRH-A at 4 μg/gbw induced a significantly higher response than 2 and 6 μg/gbw GnRH-A, 5 and 10 IU/gbw hCG, and both controls but was not different from Amphiplex. There was no significant difference in sperm concentration between 6 μg/gbw GnRH-A and the treatments with 2 μg/gbw GnRH-A and Amphiplex and the controls. Similarly, no difference was found among 2, 6 μg/gbw GnRH-A and Amphiplex, while 2, 4 μg/gbw GnRH-A and Amphiplex were significantly different to 5 and 10 IU/gbw hCG and both controls (Table 2 and Table 3).

### 3.2. Hormonally Induced Sperm Concentration–Response Curves

**Figure 4:**
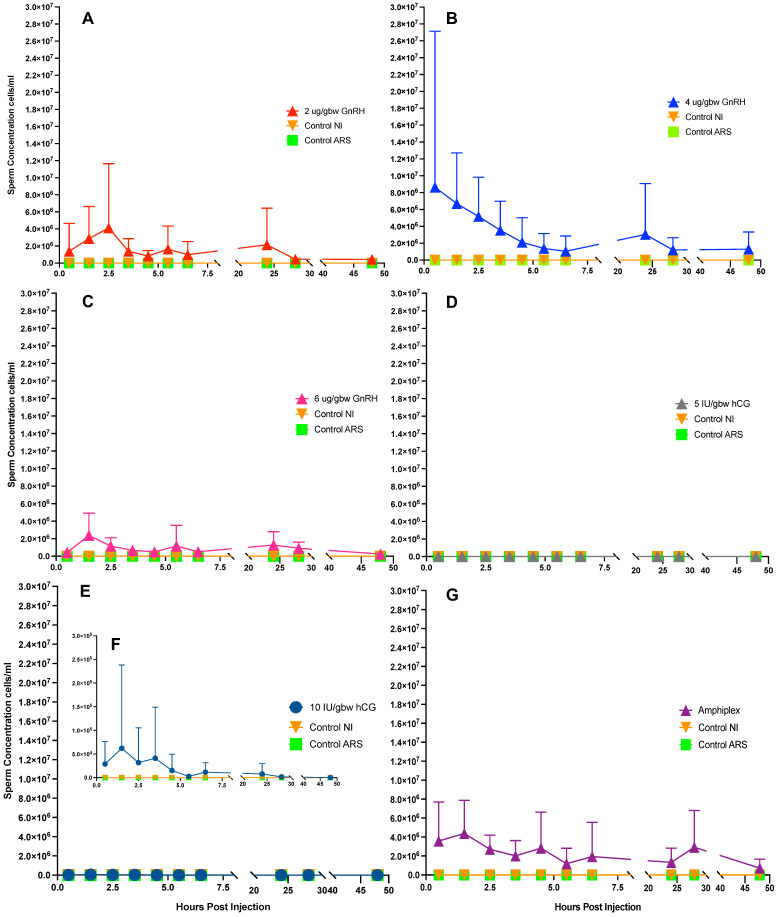
Time and dose-dependent sperm concentration–response curves post hormone (*n* = 7 per treatment) and two control treatments (no hormone, *n* = 4 per treatment). Sperm concentration values for all time points were expressed as mean ± SD over 0.5, 1.5, 2.5, 3.5, 4.5, 5.5, 6.5, 24, 28 and 48 h post-injection: (**A**) 2 μg/gbw GnRH-A, (**B**) 4 μg/gbw GnRH-A, (**C**) 6 μg/gbw GnRH-A, (**D**) 5 IU/gbw hCG, (**E**) 10 IU/gbw hCG, (**F**) 10 IU/gbw hCG at a lower scale for visualization and (**G**) Amphiplex.

Sperm concentration curves show that treatment with 4 μg/gbw GnRH-A and Amphiplex stimulate a higher early response compared to all other treatments, producing an average of 4.37 × 10^6^ ± 2.42 × 10^6^ and 2.91 × 10^6^ ± 7.92 × 10^5^ cells/mL during sperm production peaks respectively (Figure 4B,G). Treatment with 2 and 6 μg/gbw GnRH-A stimulated the production of spermic urine at 0.5 h post-injection (hpi) at lower concentrations (1.38 × 10^6^ ± 3.28 × 10^6^, 4.39 × 10^5^ ± 3.15 × 10^5^) (Figure 4A,C). No sperm was obtained after stimulation with 5 IU/gbw hCG at any of the time points (Figure 4D), while 10 IU/gbw hCG elicited a mild response, starting at 0.5 h post-injection (2.89 × 10^4^ ± 4.74 × 10^4^) (Figure 4E,F). Control treatments did not stimulate the production of spermic urine (Figure 4A–F).

Response to stimulation with GnRH-A (all concentrations) and Amphiplex remained high at 24 h (range: 1.28 × 10^6^ ± 1.53 × 10^6^ to 3.04 × 10^6^ ± 6.03 × 10^6^) and 28 h (range: 4.55 × 10^5^ ± 3.18 × 10^5^ to 2.92 × 10^6^ ± 3.88 × 10^6^) post-injection, with Amphiplex stimulating the production of higher concentrations at this time point (2.92 × 10^6^ ± 3.88 × 10^6^). While still present, the response to stimulation with 10 IU/gbw hCG was lower compared to GnRH-A and Amphiplex treatments at both 24 h (7.7810^3^ ± 2.20 × 10^4^) and 28 h (1.67 × 10^3^ ± 4.71 × 10^3^) post-injection. At 48 h post-injection, 4 μg/gbw GnRH-A showed a good response (1.30 × 10^6^ ± 2.04 × 10^6^), followed by Amphiplex (7.14 × 10^5^ ± 9.57 × 10^5^), 2 μg/gbw GnRH-A (4.31 × 10^5^ ± 4.58 × 10^5^), 6 μg/gbw GnRH-A (2.55 × 10^5^ ± 2.83 × 10^5^) and 10 IU/gbw hCG (no response) (Figure 4A–F).

### 3.3. Duration of Hormonal Stimulation Response

GnRH-A was the most efficient elicitor of sperm responses compared to hCG and Amphiplex. At 2 μg/gbw, GnRH-A induced the most prolonged response (continuous, 6 h peak from 0.5 to 6.5 h pi, still showing from 24 to 28 h pi). GnRH-A at 4 μg/gbw had similar effects but with a slightly shorter response (discontinuous, 5 h peak—from 0.5 to 5.5 h and a 1 h peak at 24 h pi), while GnRH-A 6 μg/gbw had a slight delay in the initiation of a response (discontinuous, 2 h peak—from 1.5 to 3.5 h pi, a 1 h peak at 5.5 h, then at 24 and 28 h pi). At the lower concentration of 5 IU/gbw, hCG was unsuccessful in stimulating any sperm production. However, 10 IU/gbw hCG showed similar but shorter chronological patterns of sperm induction (continuous, 6 h peak—from 0.5 to 6.5 h pi and a 1 h peak at 24 h pi) than that of 2 μg/gbw GnRH-A, while Amphiplex showed a discontinuous, 4 h peak (from 0.5 to 4.5 h pi, a 1 h peak at 6.5 h pi and a 1 h peak at 28 h pi) (Table 4).

### 3.4. Effect of Hormonal Treatments on Sperm Quality Parameters

**Figure 5:**
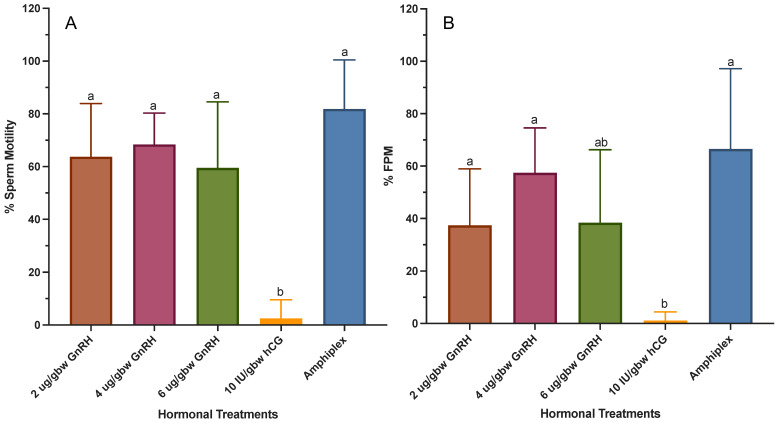
Percentage sperm motility (**A**) and sperm forward progressive motility (FPM; (**B**)) during sperm production peaks after stimulation with hormonal treatments (*n* = 7 per treatment). Significant differences in treatments are indicated by the differential lettering. Values for all data were expressed as mean ± SD. Means were considered statistically different if *p* < 0.05.

All treatments, with the exception of 10 IU/gbw hCG (~2.50 ± 7.07%), stimulated the production of sperm with percentage motility >59% (*W*_(4,14.20)_ = 61.56, *p* < 0.0001), with Amphiplex (81.86 ± 18.58%) and 4 μg/gbw GnRH-A (68.39 ± 11.90%) showing a higher percentage motility than the rest of the treatments (Figure 5A). Forward progressive motility of spermatozoa also differed among treatments (W_(4,16.27)_ = 36.75, *p* < 0.0001), with Amphiplex (66.52 ± 30.64%) and GnRH-A at 4 μg/gbw (57.44 ± 17.14%) presenting a higher percentage FPM, followed by 6 and 2 μg/gbw GnRH-A (38.41 ± 27.82% and 37.39 ± 21.57%, respectively), and 10 IU/gbw hCG (1.11 ± 3.33%) (Figure 5B). These results indicate that Amphiplex and 4 μg/gbw GnRH-A stimulate the production of sperm with the best motility parameters.

**Figure 6:**
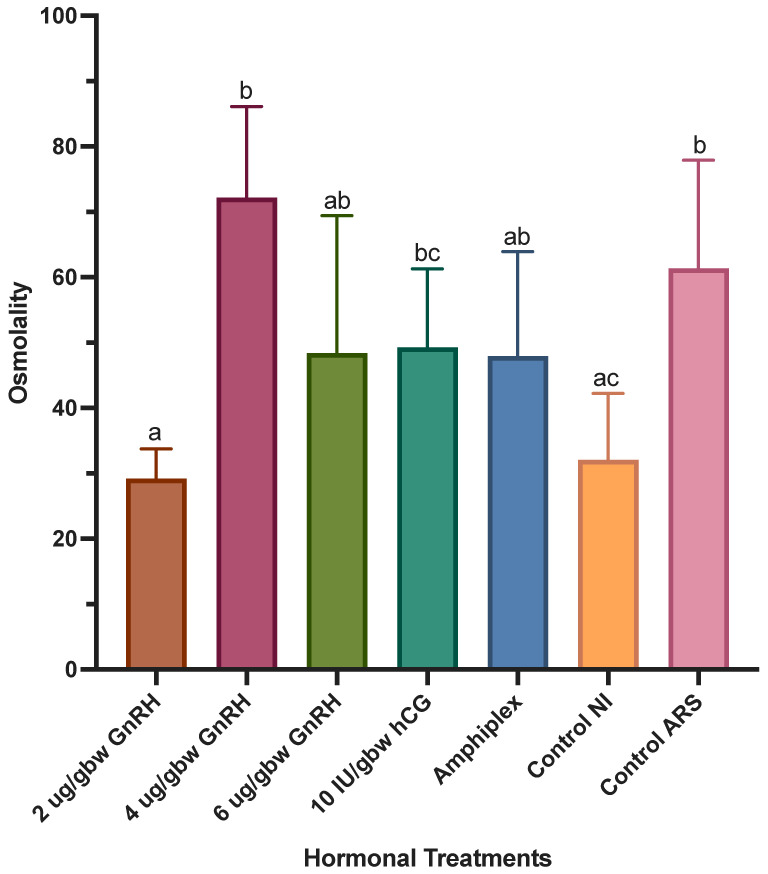
Osmolality of spermic urine during sperm production peaks after stimulation with hormonal treatments (*n* = 7 per treatment) and two control treatments (no hormone, *n* = 4 per treatment). Significant differences in treatments are indicated by the differential lettering. Values for all data were expressed as mean ± SD. Means were considered statistically different if *p* < 0.05.

There was a significant effect of hormonal treatments on spermic urine osmolality (*W*_(6,19.57)_ = 15.41, *p* < 0.0001) (Figure 6). Treatment with 2 μg/gbw GnRH-A (29.15 ± 4.54%) showed significantly lower spermic urine osmolality than 4 μg/gbw GnRH-A (72.16 ± 13.97%), 10 IU/gbw hCG (49.29 ± 11.99%) and the control with ARS (61.38 ± 16.54%). Treatment with 4 μg/gbw GnRH-A showed a higher spermic urine osmolality than that of the control with no injection (NI, 32.07 ± 10.15%) and a higher osmolality compared to the rest of the treatments while showing no difference with the ARS control. There was no significant difference among 2, 6 μg/gbw GnRH-A, Amphiplex and the control with no injection, nor among 4, 6 μg/gbw GnRH-A, 10 IU/gbw hCG, Amphiplex and the control with ARS. Nearly significant differences were observed between 4 μg/gbw GnRH-A and 10 IU/gbw hCG (*p* = 0.0863) and between 10 IU/gbw hCG and the control with no injection (*p* = 0.0964).

**Figure 7:**
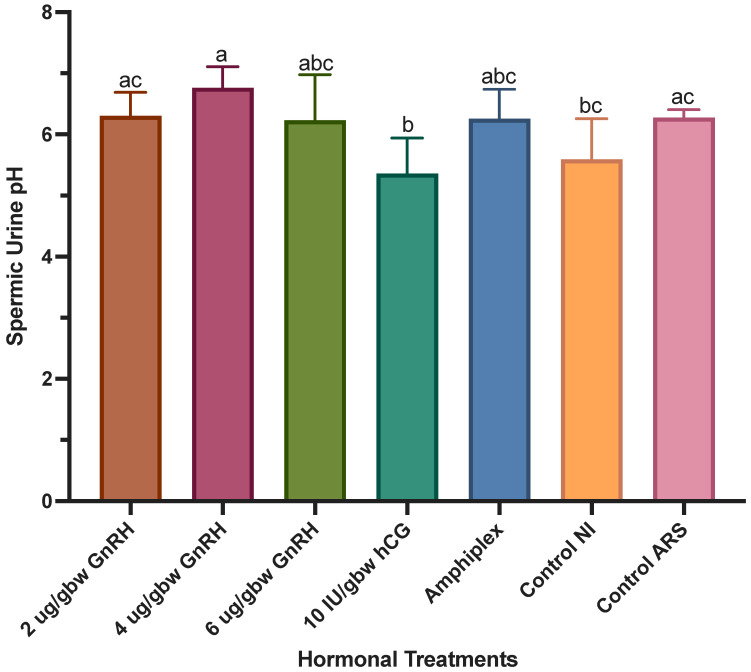
Spermic urine pH measured during sperm production peaks after stimulation with hormonal treatments (*n* = 7 per treatment) and two control treatments (no hormone, *n* = 4 per treatment). Significant differences in treatments are indicated by the differential lettering. Values for all data were expressed as mean ± SD. Means were considered statistically different if *p* < 0.05.

Spermic urine pH differed among hormonal treatments (*W*_(6,18.90)_ = 6.33, *p* = 0.0009) (Figure 7). The pH of spermic urine obtained after stimulation with 4 μg/gbw GnRH-A was significantly higher (6.76 ± 0.34) than that of 10 IU/gbw hCG (5.36 ± 0.57) and the urine obtained from controls with no injection (5.59 ± 0.66). On the other hand, 10 IU/gbw hCG showed a significantly lower spermic urine pH than that of 2 μg/gbw GnRH-A (6.30 ± 0.38) and the control with ARS (6.27 ± 0.12). There was no difference in pH of spermic urine obtained after treatment with 2, 4, 6 μg/gbw GnRH-A, Amphiplex and the control with ARS (range: 6.23 ± 0.74 to 6.76 ± 0.34), as well as among 2, 6 μg/gbw GnRH-A, Amphiplex and both controls (range: 5.59 ± 0.66 to 6.25 ± 0.48). There was no difference between the controls, although the control with ARS showed a higher, but not significantly different pH than that of the control with no injection.

### 3.5. Morphological Assessment

**Figure 8:**
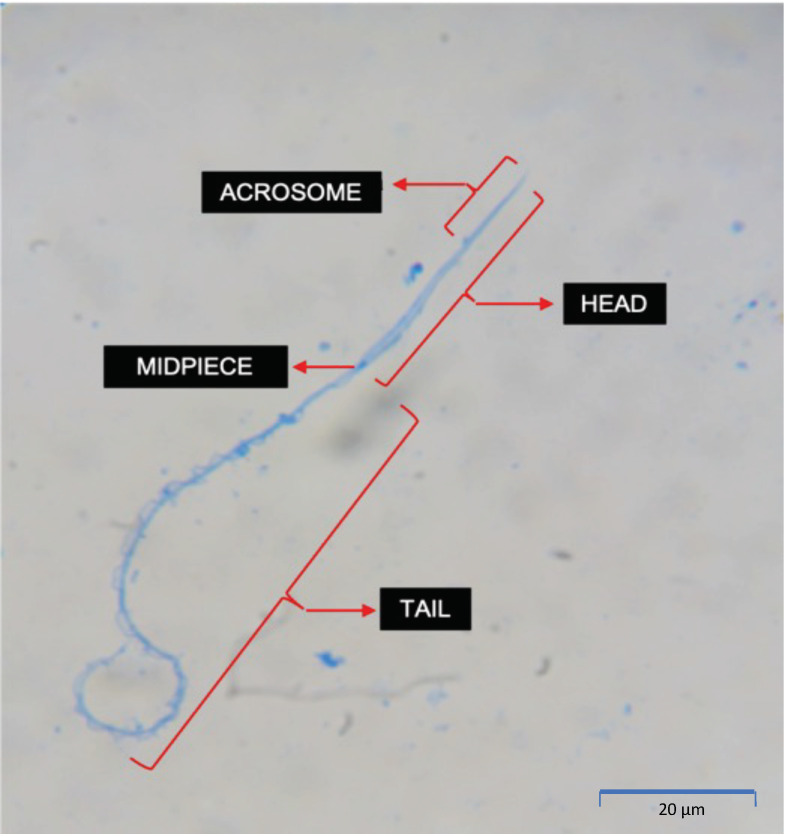
Micrograph of *Craugastor evanesco* normal spermatozoa stained with Coomassie Blue (1000× magnification) showing the main structural features.

**Figure 9:**
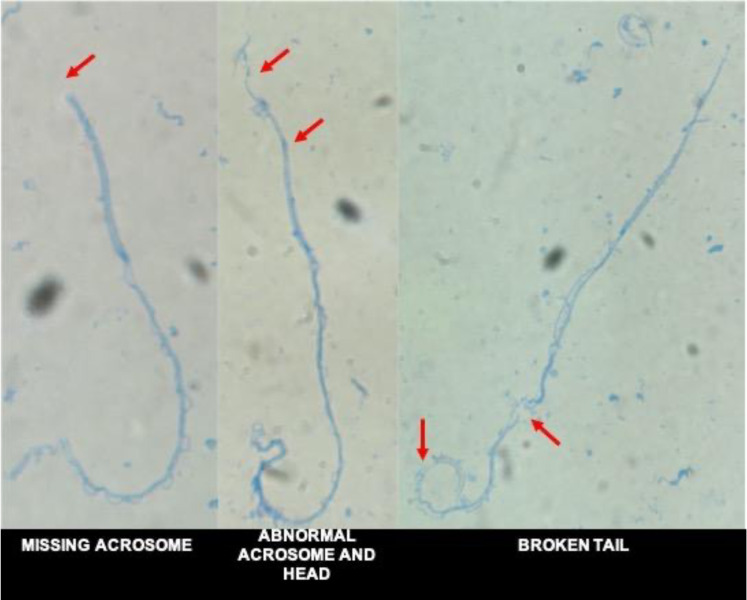
Micrograph of *Craugastor evanesco* spermatozoa stained with Coomassie Blue (1000× magnification) showing the most common morphological abnormalities (arrows).

*Craugastor evanesco* spermatozoa have a filiform shape (Figure 8). A pointy and elongated acrosome is located at the anterior part of the head. A small midpiece located at the base of the head connects the head with a long tail that exhibits two filaments joined together by an undulating membrane. The average size of the head and tail are 39.75 ± 2.73 μm and 105.91 ± 9.15 μm, respectively. Cells with abnormal morphology (abnormal or missing acrosome, abnormal head and broken or coiled tail) were observed in all samples (Figure 9).

**Figure 10:**
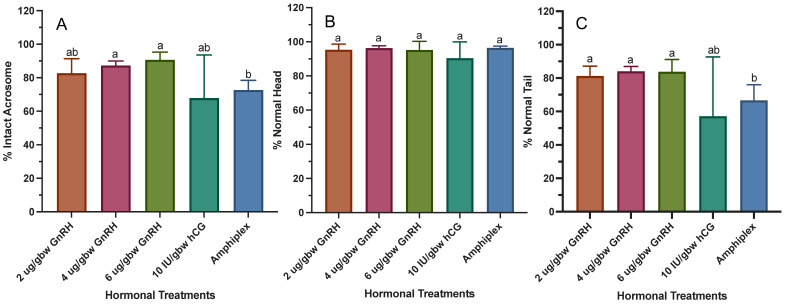
Percentage of normal spermatozoa by morphological feature after stimulation with hormonal treatments (*n* = 7 per treatment). (**A**) Intact acrosome, (**B**) normal head and (**C**) normal tail. Values for all data were expressed as mean ± SD. Bars with different letters are statistically different (*p* < 0.05).

To understand if there is an effect of hormonal treatments on morphological parameters, three different traits were examined: acrosome, head and tail integrity (Figure 10). All hormonal treatments showed percentages of acrosome integrity higher than 67% (range: 67.83 ± 25.72% to 90.66 ± 4.63%) (Figure 10A). All three GnRH-A treatments produced a percentage of intact acrosomes higher than 80% (range: 82.72 ± 8.64% to 90.66 ± 4.63%). There was a significant effect in the percentage of intact acrosomes among hormonal treatments (*W*_(4,14.92)_ = 10.94, *p* = 0.0002) with Amphiplex showing a significantly lower percentage of intact acrosomes (72.63 ± 5.82) than 4 and 6 μg/gbw GnRH-A (87.32 ± 2.64 and 90.66 ± 4.63, respectively), while not differing from 2 μg/gbw GnRH-A and 10 IU/gbw hCG. Although treatment with 10 IU/gbw hCG showed no statistical difference due to its larger SD, a lower acrosome integrity than that of the other treatments was noticeable.

Hormonal treatments had no effect on the percentage of normal heads (Figure 10B). All treatments showed percentages of normal heads >90% (range: 90.38 ± 9.57% to 96.41 ± 1.08%). As with acrosome integrity, hormonal treatments did have an effect on tail integrity (*W*_(4,14.35)_ = 5.89, *p* = 0.0051) (Figure 10C). Treatment with Amphiplex showed a lower percentage of intact tails (66.63 ± 9.38%) compared to all GnRH-A treatments (range: 81.23 ± 5.89% to 84.03 ± 2.92%) but was not different from 10 IU/gbw hCG (57.15 ± 35.53). As observed in the analysis of acrosome integrity, 10 IU/gbw hCG showed no statistical difference due to its larger SD but a lower tail integrity than that of the other treatments was evident.

**Figure 11:**
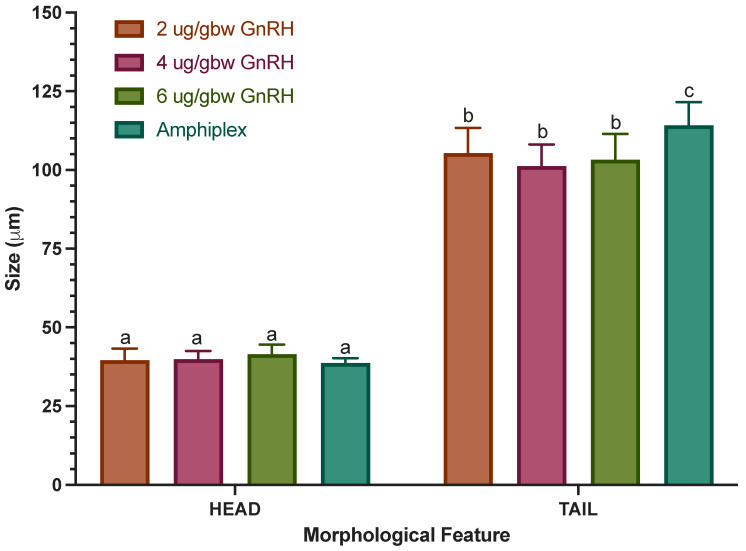
Average size (in μm) of head and tail of *C. evanesco* spermatozoa by hormonal treatment (*n* = 100). Values for all data were expressed as mean ± SD. Bars with different letters are statistically different (*p* < 0.05).

An analysis of size (head and tail) indicated that there was a significant main interaction effect between size and hormonal treatments (F_(3,190)_ = 20.13, *p* < 0.0001), suggesting that the size of a spermatozoon’s head and tail are hormonally dependent (Figure 11). Additionally, there was a main simple effect of hormonal treatments (F_(3,190)_ = 12.61, *p* < 0.0001). Due to the limited number of spermatozoa obtained with the 10 IU/gbw hCG treatment, we were not able to include it in this analysis. Our results indicate that treatment with Amphiplex stimulated the production of spermatozoa with longer tails (114.21 ± 7.39) compared to all other treatments (*p* < 0.0001).

## 4. Discussion

This study is the first in reporting the successful sperm collection and characterization of captive *C. evanesco* after hormonal stimulation. This is a recently described species for which there is little to no information available. Therefore, our results contribute to new and important knowledge on the reproductive physiology of this species.

### 4.1. Sperm Concentration Post Hormone Induction

The effectiveness of hormonal treatments in stimulating the production of *C. evanesco* spermic urine varied depending on the hormone and concentration used, supporting once again that responses to hormonal stimulation are species-specific and vary among species or genera [10,24]. GnRH-A and Amphiplex were the most effective treatments in retrieving highly concentrated spermic urine samples, as observed before in species such as *Atelopus zeteki*, *Anaxyrus americanus*, *A. baxteri* and *Pseudophryne corroboree* [12,18,25].

Briefly, the reproductive cycle of anurans, as that of most tetrapods, is under the hierarchic control of the HPG axis (Hypothalamic–Pituitary–Gonadal), regulated by the production of Gonadotropin-Releasing Hormone (GnRH). GnRH is a decapeptide produced by GnRH neurosecretory cells and it is released into the portal capillary vessels. GnRH acts on the anterior lobe of the pituitary, stimulating the production of Luteinizing Hormone (LH) and Follicle-Stimulating Hormone (FSH). LH and FSH act on the gonads stimulating the production of sex steroids and gametogenesis [14,26,27]. In amphibians, spermiation results from the stimulation of Leydig and Sertoli cells in the testes after an LH and/or FSH surge, respectively [28].

In our study, a concentration-dependent response was observed in all GnRH-A treatments, where 4 µg/gbw GnRH-A stimulated the production of more than double the sperm concentration obtained with 2 and 6 µg/gbw GnRH-A. This could be explained by the fact that the physiological responsiveness to GnRH, in terms of the number of GnRH receptors in the cell membrane of GnRH neurons and gonadotrophs and the secretion of LH and FSH, is regulated mainly by GnRH itself [29]. The activation of GnRH receptors is dose-dependent, with low doses of GnRH agonists causing down-regulation or inhibition of the GnRH-1 receptors and a negative feedback loop associated with no or low LH release, whereas higher optimal doses increase neuronal firing rates and higher LH surges [29,30,31].

GnRH-A 2 µg/gbw appears to be a sub-physiological concentration, not strong enough to elicit a physiological response in *C. evanesco* compared to that of 4 µg/gbw GnRH-A. On the other hand, 6 µg/gbw GnRH-A appears to be on the verge of the supraphysiological concentration for this species. Supraphysiological concentrations of GnRH have been shown to produce desensitization or downregulation of the GnRH receptors, pituitary inhibition and/or suppression of testosterone production due to LH and FSH receptor reduction in the testes [32], capable of eliciting a contraceptive effect, explaining the lower response obtained after this treatment. In other species such as *Litoria booroolongensis*, treatment with 2 µg/gbw GnRH-A shows a nearly significantly higher response than that of 4 µg/gbw GnRH-A, while the lowest concentration of 0.5 µg/gbw GnRH-A produced the best results in this species [33], suggesting that the threshold for subphysiological, physiological and supraphysiological doses may vary among species. Taken together, these findings suggest that GnRH-A at 4 µg/gbw acts as an optimal physiological dose for *C. evanesco*. Studies on hormonal stimulation in captive *Atelopus zeteki* [12] and wild *Agalychnis callidryas* [34] have shown 4 µg/gbw GnRH-A to be the optimal GnRH-A concentration for the collection of high-quality spermic urine in these species as well.

Contrastingly to GnRH-A, Human Chorionic Gonadotropin (hCG) circumvents the HPG axis and acts directly on the gonads, binding to the same receptors as LH on the Leydig cells [35]. Our results suggest that treatment with the tested concentrations of hCG had little to no effect on stimulating the production of spermatozoa in *C. evanesco*; the lower concentration of 5 IU/gbw did not elicit a response at all, whereas an incipient response was observed after stimulation with a higher concentration (10 IU/gbw). Several studies in anurans have shown highly variable responses to hCG stimulation with fewer species responding successfully to this treatment compared to GnRH-A (*Xenopus laevis*, *Silurana tropicalis*, *Litoria chloris*, *Litoria aurea*, *Anaxyrus fowleri*, *Epidalea calamita*, *Rhinella marina*, *Heleioporus eyrei*, *Neobatrachus pelobatoides*, *Litoria booroolongensis* and *Litoria verreauxii alpina*); others show no response or respond poorly to hCG (*Pseudophryne corroboree* and *Geocrinia rosea*), while some respond positively to both hormones, with GnRH-A showing a better response (*Atelopus zeteki*, *Rana temporaria* and *Crinia pseudinsignifera*) [11,21,33,36,37,38,39]. Previous studies in *Rhinella arenarum* suggested a more efficient role of FSH than that of LH and hCG in stimulating spermiation, showing a differential sensitivity, dose-dependent response to these different gonadotropins and an FSH receptor-mediated response with higher affinity for FSH than for LH and hCG [28]. One explanation for the lack of response to this treatment in this species is that spermiation is possibly stimulated by both LH and FSH via their receptors, as observed in *Rana temporaria* and *Lithobates pipiens* [40], and as hCG stimulates LH receptors only, showing a lower affinity for FSH receptors, the response is absent, incomplete and incipient. Adding to this, it is known that the *β* subunit of hCG appears to be less compatible with LH receptors than the LH *β* subunit. These explanations would support why GnRH-A is more efficient in stimulating sperm production than hCG since GnRH-A stimulates the production of endogenous LH as well as FSH and both gonadotropins are acting in the testes. Exploring the possibility of utilizing higher hCG concentration may be necessary to better understand if the lack of or incipient response to hCG in this species was due to a sub-physiological dose response or due to low affinity to an FSH receptor-mediated response.

Although not significantly different from any of the GnRH-A treatments, Amphiplex showed the second-best response at stimulating the production of highly concentrated sperm samples in this species, despite the fact that the GnRH-A concentration in this treatment is 5, 10 and 15 times lower than that of 2, 4 and 6 µg/gbw GnRH-A treatments, respectively. Despite some contradictory evidence, previous studies in different taxonomic groups, including amphibians, suggest a possible inhibitory action of the central dopaminergic system on the biosynthesis of gonadotropins. Dopamine, through its DA-2 receptors in the pituitary, might exert a regulatory role on the GnRH and GnRH receptor gene expression, directly impacting LH and FSH release [11,19,41,42]. A previous study from our research group showed Amphiplex successfully stimulated the production of high-quality spermic urine in the Panamanian Golden Frog (*Atelopus zeteki*) with a GnRH-A concentration 10 times lower than that of the best-working GnRH-A treatment [12]. Other amphibian species such as *Lithobates pipiens*, *Ceratophrys ornata*, *Ceratophrys cranwelli* and *Odontophrynus americanus* have shown a positive spawning and breeding behavior response as well as a biphasic increase in the gene expression of *lhb*, *fshb* and *gnrh1* over time after treatment with Amphiplex higher than that observed with GnRH-A alone [17,43], suggesting that metoclopramide might potentiate the effect of lower doses of GnRH-A. Taken together, our results indicate that the optimal treatment for the collection of highly concentrated sperm samples of *C. evanesco* is 4 µg/gbw GnRH, followed by Amphiplex and 2 µg/gbw GnRH as sub-optimal treatments and finally, 6 µg/gbw GnRH, 5 and 10 IU/gbw hCG as non-optimal treatments.

### 4.2. Hormonally Induced Sperm Concentration–Response Curves

All samples were collected over a period of 10 time points, starting at 0.5 h pi and ending at 48 h pi. At the 0.5 h pi time point, all treatments, except for 5 IU/gbw hCG, stimulated the production of spermatozoa, as observed in the Panamanian Golden Frog at the same time point [12]. The samples obtained with the optimal treatment of 4 µg/gbw GnRH at 0.5 h pi (8.64 × 10^6^ ± 1.90 × 10^7^ cells/mL) contained 6.2-, 19.6-, 298.9- and 2.4-fold higher sperm concentration than 2, 6 µg/gbw GnRH-A, 10 IU/gbw hCG and Amphiplex, respectively, at the same time point, gradually decreasing until reaching the lowest point of the first peak at 6.5 h pi, still producing sperm concentrations in the order of 10^6^ cells/mL. The efficacy of the 4 µg/gbw GnRH-A was such that the highest sperm concentration obtained during peaks was produced by the first time point (0.5 h pi). A study in *Rana catesbiana* provided evidence of a rapid (10 min) GnRH dose-dependent LH and FSH concentration increase in circulating blood after GnRH-A stimulation, followed by a spermiation response 30 to 45 min post-stimulation [44]. Obringer et al. detected elevated LH concentrations in plasma from 15 to 30 min after GnRH-A stimulation in *Anaxyrus americanus* [25]. This rapid effect of GnRH agonists in anuran testicular tissue might promote cell division and spermiation in a dose and post-stimulation time-dependent manner [45]. The rapid and high response observed in the 4 µg/gbw GnRH-A treatment may be related to a rapid LH and FSH surge and their effect on Leydig cell androgen production, optimal swelling of Sertoli cells, release of mature spermatozoa into the lumen of seminiferous tubules and, finally, on spermiation. With the exception of 5 IU/gbw hCG, all other treatments showed similarities in their sperm production patterns, with initial lower concentrations at 0.5 hpi, reaching their first peak concentrations between 1.5 (6 µg/gbw GnRH-A, 10 IU/gbw hCG and Amphiplex) and 2.5 hpi (2 µg/gbw GnRH-A) followed by a drop and subsequent increases. The slower response observed in other GnRH-A treatments compared to that of 4 µg/gbw GnRH-A might be due to a dose-dependent latency in the LH/FSH surge [44], insufficient release of these gonadotropins during the late surges and an incomplete or partial response from all further gonadotropin-dependent physiological mechanisms required for successful spermiation.

Initially, collection time points for this species were set up to 24 hpi. Due to the high sperm concentrations collected (10^6^ cells/mL) from most treatments at this time point, two more time points (28 and 48 hpi) were added to learn how long the hormone treatment effects lasted in this species. Out of the five treatments that were successful in stimulating sperm production, only one (10 IU/gbw hCG) failed to produce any spermic urine at 48 hpi but still produced spermic urine at low concentrations (10^4^) at the 28 hpi time point. The optimal treatment of 4 µg/gbw GnRH-A was the only one to maintain the production of spermic urine with concentrations in the order of magnitude of 10^6^ cells/mL at 48 hpi, followed by Amphiplex, 2 µg/gbw GnRH-A and 6 µg/gbw GnRH-A with concentrations of 10^5^ cells/mL (listed in a descendent manner). To our knowledge, most studies but one [18] on the duration of the effect of hormonal stimulation in the production of spermic urine in several anuran species have assessed up to 24 hpi [12,21,25,34,36,46,47]. From these, a dose-dependent and species-specific decrease in sperm production is evident, with some producing no sperm at all and others still producing a fair number of spermatozoa after stimulation. A study by Vu and Trudeau assessed the gene expression of *lhb*, *fshb* and *gnrh1* after treatment with GnRH-A in female *Lithobates pipiens,* finding a biphasic response to stimulation with lower doses of this hormone alone and with 0.4 µg/gbw GnRH-A+10 µg/gbw Metoclopramide at 12, 24 and 36 hpi [42]. With GnRH-A alone and GnRH-A+Metoclopramide, gene expression initially increased at 12 hpi, decreased at 24 hpi and increased significantly again at 36 hpi, with GnRH-A+Metoclopramide showing the highest increases. Even though it is known that responses to exogenous hormones differ between sexes, these results support our findings of prolonged responses over 48 hpi and the presence of late peaks after stimulation with these treatments in *C. evanesco*.

All treatments differed in their sperm production peak pattern and duration. Two of the treatments showed two different sperm production peaks (4 µg/gbw GnRH-A and 10 IU/gbw hCG), two treatments showed three different sperm production peaks (2 µg/gbw GnRH-A and Amphiplex) and one treatment showed four sperm production peaks (6 µg/gbw GnRH-A) over the 10 collection time points. Other studies have reported different sperm production peaks using similar hormones, similar or different concentrations, time points and species. Obringer et al., first measured sperm concentration in *Anaxyrus americanus* after stimulation with ~0.18 µg/gbw GnRH-A (they reported a final concentration of 4 µg per animal and a minimum weight of 22 g per animal) at 3, 7 and 12 hpi, with the peak sperm concentration produced at 7 hpi. Subsequently, they tried the same time points at a lower GnRH-A concentration of ~0.04 µg/gbw (final dose of 1 µg) and observed a sperm production peak at 12 hpi [25].

Another study compared the same time points of 3, 7 and 12 hpi in eight different species using the concentrations of 2 µg/gbw GnRH-A and 13 IU/gbw of hCG [38]. After the GnRH-A treatment, their results showed that three of the species (*Crinia georginia*, *Heleioporus albopunctatus* and *Neobatrachus pelobatoides*) peaked at 7 hpi, two other species (*Pseudophryne guentheri* and *Geocrinia rosea*) peaked at 3 hpi, one species (*Helioporus eyrei*) peaked at 12 hpi and two species (*Crinia pseudinsignifera* and *Crinia glauerti*) showed two different separated peaks at 3 and 12 hpi and 3 and 7 hpi, respectively. As for the hCG treatment, three species (*Crinia glauerti*, *Crinia georginia* and *Neobatrachus pelobatoides*), two of which coincided with the GnRH-A treatment, showed a sperm production peak at 7 hpi, four species (*Crinia pseudinsignifera, Geocrinia rosea*, *Pseudophryne guentheri* and *Heleioporus eyrei*), one of which coincided with the GnRH-A treatment, peaked at 12 hpi and one species (*Heleioporus albopunctatus*) showed two separate peaks at 3 and 12 hpi. These studies utilized the same collection time points with a significant time gap between them that could have missed one or more peaks; nevertheless, at least one peak was identified and species-specific hormone concentration and time-dependent differences were evident.

### 4.3. Duration of Hormonal Stimulation Response

As observed in *C. evanesco* and *Atelopus zeteki* [12], hormonal treatments can produce more than one sperm concentration peak over time and these peaks can appear as early as 0.5 hpi. When comparing hormone concentrations and responses in *C. evanesco* with those observed in *A. zeteki*, similar hormones and concentrations were utilized, and differences in the type of response were noted, with the most apparent being the number and duration of sperm production peaks. The longest continuous peak observed in *C. evanesco* lasted 6 h (2 µg/gbw GnRH-A) and all treatments showed at least two peaks, while in *A. zeteki* the longest peak lasted 2.5 h (10 IU/gbw hCG) and only one treatment showed more than one peak (2 µg/gbw GnRH-A), although it is important to note that collection time points did not exceed 24 hpi in this species. Taken together, these results suggest a species, hormone, concentration and time-dependent pattern in sperm production peaks. Spermatogenic cycles inside testicular cysts (intra-cystic) are mostly synchronous in anurans; nevertheless, some studies have shown the existence of spermatogenic intracystic asynchronicity in some species [48,49]. A possible explanation for the presence of more than one sperm production peak could be intracystic asynchronicity, where different cysts are at different spermatogenic stages [21,50] at a given time, causing an asynchronous release of high concentrations of mature spermatozoa after hormonal stimulation once the cycle is completed (i.e., peaks). This, coupled with variations in the swelling response of Sertoli cells that promotes the expulsion of mature spermatozoa from these cells into the lumen [51] after hormonal stimulation, could explain the presence of sperm concentration peaks.

The occurrence of one or more sperm concentration peaks and the duration-dependence of these after hormonal stimulation could be the result of several factors acting simultaneously: (1) the species; (2) the hormone type and concentration, which will stimulate receptors in a sub-optimal, optimal or supra-optimal manner [52]; (3) time and dose-dependent biphasic response of gonadotropin gene expression to hormonal stimulation [42]; (4) species-specific GnRH-A, LH and FSH circulating half-life; (5) the response of the target tissues of these hormones: LH and FSH sub-optimal, optimal or supra-optimal surge (for GnRH) or androgen concentrations (for hCG); (6) the species intracystic or intercystic synchrony or asynchrony of spermatogenic stages [53]; (7) the species duration of the different spermatogenic stages, where some of the cysts might contain more mature Sertoli cells than others that are delayed and responding later to the hormonal stimulation [21]; (8) the number of testicular cysts with mature spermatozoa ready for release [53]; (9) the effect of sub-optimal, optimal or supra-optimal gonadotropin concentrations on the required swelling of Sertoli cells to promote adequate expulsion of most mature spermatozoa into the lumen of seminiferous tubules [21]; (10) selection of optimal collection time points [10] and (11) adequate assessment of samples per time point. Given the time points reported in this study, it is possible that additional peaks were missed, or the continuity of a single time point may not have been detected between 6.5, 24, 28 and 48 hpi. Therefore, further research is necessary to clarify these possible gaps and fully understand the testicular cycle in *C. evanesco*, critical to the future development of other assisted reproductive technologies such as sperm cryopreservation or the synchronization of breeding events.

### 4.4. Effect of Hormonal Treatments on Sperm Quality Parameters

During the sperm production peaks, all treatments, with the exception of 10 IU/gbw hCG, produced spermic urine with percentage motility in a range between 59 and 82%. Although not significantly different, Amphiplex showed the highest percentage of motile sperm, followed by 4 µg/gbw GnRH-A. Across treatments, the percentage FPM (<66%) was lower than the percentage motility as has been observed in other amphibian species [12,21,36,46,54]. A similar pattern was evident with Amphiplex showing the highest percentage FPM, followed by 4 µg/gbw GnRH-A. Percentage sperm motility was elevated and very similar among GnRH-A treatments; however, percentage forward progressive motility showed evident but not significant differences, with 4 µg/gbw GnRH-A producing spermic urine samples with the highest percentage FPM. In contrast, treatment with hCG showed the lowest motility parameters when compared to all treatments (~2.5% motility and ~1.1%FPM). A study on *Rana temporaria* showed that a dose of 11.7 IU/gbw hCG produced lower motility parameters than GnRH-A treatments. Although not significant, a higher concentration of the hormone increased motility parameters over a period of 24 hpi [21]. Other studies have shown positive motility response after treatment with hCG, as is the case of *Anaxyrus baxteri*, reaching motility percentages of ~80% [54]. Interestingly, species that respond well to stimulation with hCG in terms of concentration also show elevated motility parameters, as is the case of *Rhaebo guttatus* [46], *Litoria booroolongensis* [55], *Anaxyrus fowleri* [56] and *Atelopus zeteki* [12]. Some studies on fish reported a negative effect of hCG on sperm quality parameters over time, specifically on percentage motility or motility duration, compared to treatments with GnRH-A alone or GnRH-A+DA antagonists [57,58,59]. These findings support that the tested hCG concentrations used in this study negatively affect motility parameters in *C. evanesco*, drastically reducing the sperm quality in this species, also evidenced by our sperm concentration assessment. In addition to hormonal effects, it is possible that the lower FPM percentages observed in *C. evanesco,* compared to those of other aquatic breeders, are adequate for the species due to its terrestrial reproductive mode, where the cells need not swim to reach the eggs and FPM is only needed to penetrate the thick jelly layers of the eggs. Low testicular sperm motility parameters have also been reported for *Eleutherodactylus coqui* (Motility: 20.0 ± 4.2%, FPM: 2.5 ± 0.85%), which is a terrestrial and direct-developing species [60]. Another possibility could be that exposure to a hypoosmotic environment produced flagellar swelling, impairing their capacity for forward progression [61].

Osmolality, pH and ionic composition are some of the major factors related to motility activation and quality in amphibians and fishes [12,62,63,64]. It is widely known that hypotonic environments activate sperm motility in amphibians and freshwater fish [12,65]. Treatment with 4 µg/gbw GnRH-A and injection with ARS alone produced spermic urine with higher osmolalities compared to the control with no injection (i.e., plain urine). Interestingly, even though all hormones were previously diluted in and injected with ARS, treatments with 2, 6 µg/gbw GnRH-A, 10 IU/gbw of hCG and Amphiplex did not significantly differ from the control with no injection (all < 49.3 mOsm/kg). A slight increase was noticeable, with the exception of 2 µg/gbw GnRH-A, which showed even lower values than urine alone. When assessing the spermic urine pH, all treatments and the ARS control produced spermic urine with a pH > 6.2, but it was the 4 µg/gbw GnRH-A treatment that produced the spermic urine with the highest pH (6.7) compared to urine alone (5.5). These results indicate that treatment with 4 µg/gbw GnRH-A increases both spermic urine osmolality and pH in *C. evanesco*, favoring sperm quality.

A previous study on *Atelopus zeteki*, an aquatic breeder, found that neither hormonal treatments nor injection with ARS affected spermic urine osmolality in this species, while treatment with 4 µg/gbw GnRH-A decreased the spermic urine pH, although this decrease remained higher than pH 7.0 [11]. *Craugastor evanesco* is a riparian amphibian of the tropical rainforest that lays eggs in a terrestrial environment. The osmoregulatory apparatus of terrestrial amphibians works differently than that of biphasic species, helping them to prevent dehydration by rapid water absorption from the environment as well as water reabsorption from the bladder [27,66]. The particular osmoregulatory characteristics of this species, together with the effect of hormonal treatments, could have influenced the particular osmotic profiles obtained in this study. It is worth mentioning that during the sperm collection procedures, individuals were held in contact with water over the entire collection period (48 h). This could have helped decrease the plasma and therefore, urine osmolality, due to their fast water absorption rate, as has been observed in terrestrial amphibians that switch from a terrestrial to an aquatic habitat for the breeding season [66]. A study in *Lithobathes sylvatica*, an aquatic breeder, showed a beneficial osmolality reduction window favoring sperm motility (~185–55 mOsm/kg), with higher or lower osmolalities drastically reducing this parameter [67]. The effect of reduced osmolality in *C. evanesco* sperm motility did not appear to be as strong as in the species that breed in water, evidenced by the low spermic urine osmolality and high motility percentage of the 2 µg/gbw GnRH-A treatment and the average osmolality and low percentage motility of the 10 IU/gbw hCG treatment. Interestingly, spermic urine pH seemed to have a stronger influence on *C. evanesco* sperm motility.

In addition to osmotic pressure, spermic urine pH is an important factor that influences sperm motility in amphibians and fishes [11,62,63]. In our study, the two hormonal treatments that showed higher spermic urine pH were those that produced the highest sperm motility parameters (Amphiplex—pH 6.25 and 4 µg/gbw GnRH-A—pH 6.76), while 10 IU/gbw hCG produced sperm with almost no motility or FPM and showed the lowest spermic urine pH (5.36). Acidic external pH has been associated with low or even complete inhibition of sperm motility, possibly due to cytoplasmic acidification and further inactivation of the dynein protein in the flagellum [61]. On the other hand, an alkaline external pH seems to favor sperm motility with an apparent species-specific optimal window, where exposure to external alkaline conditions changes the internal pH benefiting motility parameters [61,63]. Further research is necessary to understand the combined effect of osmolality and pH in *C. evanesco* sperm motility and viability. Taken together, these results support species specificity in terms of sperm quality with respect to hormonal treatments, highly influenced by the reproductive mode and the physiological adaptations to the external environment.

### 4.5. Morphological Assessment

*Craugastor evanesco* has a terrestrial reproductive mode with females laying their eggs in humid sand burrows while the amplexed male excretes the spermic urine on top of the egg mass. The eggs, with a diameter of ~1.5 cm each (Figure S1), have two thick jelly layers and are deposited individually. As spermatozoa are deposited directly on the eggs in the absence of an aqueous external environment other than the urine that they are excreted in, the long and filiform morphology could serve as an adaptation that facilitates the penetration of the thick jelly layers to achieve successful fertilization (Supplementary Figure S1).

Treatment with Amphiplex showed significantly lower percentages of acrosome and tail integrity while still remaining higher than 70 and 65%, respectively, as well as significantly longer tails than the rest of the treatments. Dopamine is a natural antagonist of prolactin [68]. Metoclopramide, a component of Amphiplex, has been shown to increase the production of prolactin, causing hyperprolactinemia (high concentrations of circulating prolactin) in some instances, which has been related to increased sperm morphological abnormalities and alteration of other quality parameters in men and bulls [69]. Therefore, although still showing high-quality morphological parameters, the lower acrosomal and tail integrity observed in sperm collected after treatment with Amphiplex compared to other treatments could be related to a possible increase in prolactin, affecting these parameters in *C. evanesco* as well.

Treatment with 10 IU/gbw hCG, although not significant due to its elevated SD, showed lower percentages of morphological integrity on the three parameters compared to all other treatments, confirming once more that this treatment is not recommended for the collection of *C. evanesco* spermatozoa. On the other hand, treatment with 4 µg/gbw GnRH-A showed higher integrity percentages in all three morphological parameters (acrosome: 87.32 ± 2.64%; head: 96.33 ± 1.33%; tail: 84.03 ± 2.92%). Taken together, our results suggest that treatment with 4 µg/gbw GnRH-A was the best in terms of quantity and quality parameters for the stimulation and collection of *C. evanesco* spermatozoa.

## 5. Conclusions

The amphibian extinction crisis is causing the loss of valuable species and populations. Ex situ conservation programs and the development and application of Assisted Reproductive Technologies are efficient tools to mitigate these declines. This study produced new information on the reproductive biology of the species *C. evanesco*, as well as a successful sperm stimulation and collection protocol. Stimulation via the intraperitoneal injection with 4 µg/gbw GnRH-A using an injection volume of 10 µL/gbw, followed by sperm collection via cloacal catheterization between 0.5 and 5.5 h pi, will allow for the collection of spermic urine with motility parameters >60% and morphological integrity >80%. This new knowledge can be used to increase the reproductive success of the species, forming the base for the development of other, more advanced ARTs such as Artificial Fertilization for the production of viable offspring.

## Figures and Tables

**Table 1:** Hormones and concentrations administered to *C. evanesco* males.

Hormones	Concentrations
GnRH-A	2 μg/gbw
	4 μg/gbw
	6 μg/gbw
hCG	5 IU/gbw
	10 IU/gbw
Amphiplex	0.4 μg/gbw GnRH-A + 10 μg/gbw MET

**Table 2:** Comparison between hormonal treatments during sperm concentration peaks showing 4 μg/gbw GnRH-A as the optimal working concentration. Table shows CI-95% and *p* values obtained from the multiple comparison Tukey’s Test.

Hormones		Statistical Parameters	
Optimum Concentration	Sub-Optimum Concentration	CI-95%	*p*
4 μg/gbw GnRH-A	2 μg/gbw GnRH-A	−4.30 × 10^6^, −9.24 × 10^5^	0.0003
4 μg/gbw GnRH-A	6 μg/gbw GnRH-A	1.24 × 10^6^, 4.97 × 10^6^	<0.0001
4 μg/gbw GnRH-A	5 IU/gbw hCG	2.63 × 10^6^, 6.10 × 10^6^	<0.0001
4 μg/gbw GnRH-A	10 IU/gbw hCG	2.61 × 10^6^, 6.07 × 10^6^	<0.0001
4 μg/gbw GnRH-A	Control ARS	2.57 × 10^6^, 6.16 × 10^6^	<0.0001
4 μg/gbw GnRH-A	Control No Injection	2.57 × 10^6^, 6.16 × 10^6^	<0.0001

**Table 3:** Comparison between sub-optimal and non-optimal hormonal concentrations during sperm concentration peaks. Table shows CI-95% and *p* values obtained from the multiple comparison Tukey’s Test.

Hormones		Statistical Parameters	
Sub-Optimum Concentration	Non-Optimum Concentration	CI-95%	*p*
2 μg/gbw GnRH-A	5 IU/gbw hCG	1.28 × 10^5^, 3.36 × 10^6^	0.0258
Amphiplex	5 IU/gbw hCG	−4.64 × 10^6^, −1.17 × 10^6^	<0.0001
2 μg/gbw GnRH-A	10 IU/gbw hCG	1.04 × 10^5^, 3.35 × 10^6^	0.0295
Amphiplex	10 IU/gbw hCG	−4.61 × 10^6^, −1.15 × 10^6^	<0.0001
2 μg/gbw GnRH-A	Controls	6.93 × 10^4^, 3.45 × 10^6^	0.0358
Amphiplex	Controls	1.12 × 10^6^, 4.70 × 10^6^	0.0001

**Table 4:** Sperm concentration peaks by hormonal treatment across 10 collection time points. Different colors indicate different peaks.

Hormone	Sperm Concentration Peaks									
	Collection Time Points (Hours Post Injection)									
	0.5	1.5	2.5	3.5	4.5	5.5	6.5	24	28	48
2 μg/gpc GnRH										
4 μg/gpc GnRH										
6 μg/gpc GnRH										
5 IU/gpc hCG										
10 IU/gpc hCG										
Amphiplex										

## Data Availability

Data is unavailable due to privacy restrictions.

## Supplementary Materials

The following supporting information can be downloaded at: https://www.mdpi.com/article/10.3390/ani13172689/s1, Figure S1: Description of *C. evanesco’s* eggs.
