# Supplementary figures and images for: Recovery and Characterization of Spermatozoa in a Neotropical, Terrestrial, Direct-Developing Riparian Frog (Craugastor evanesco) through Hormonal Stimulation
Source: Animals (Basel). 2023 Aug 22;13(17):2689. doi: 10.3390/ani13172689 (PMC10486684; doi:10.3390/ani13172689)

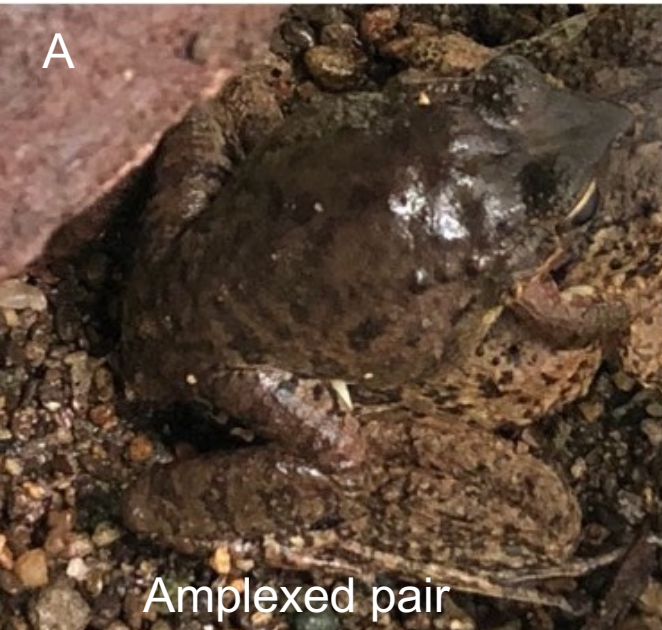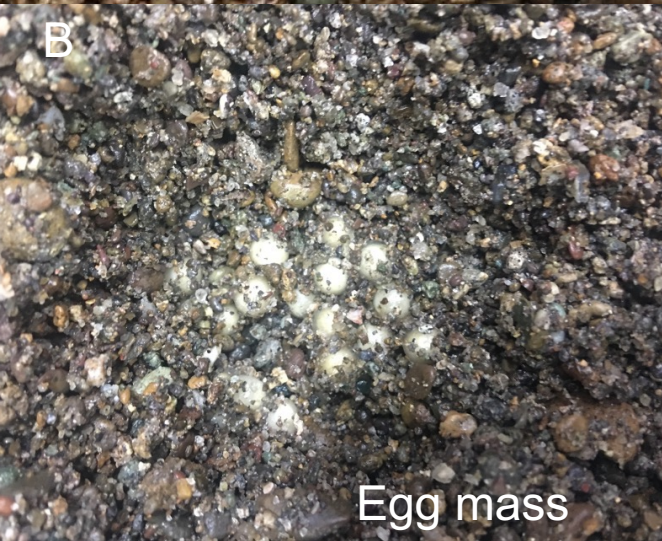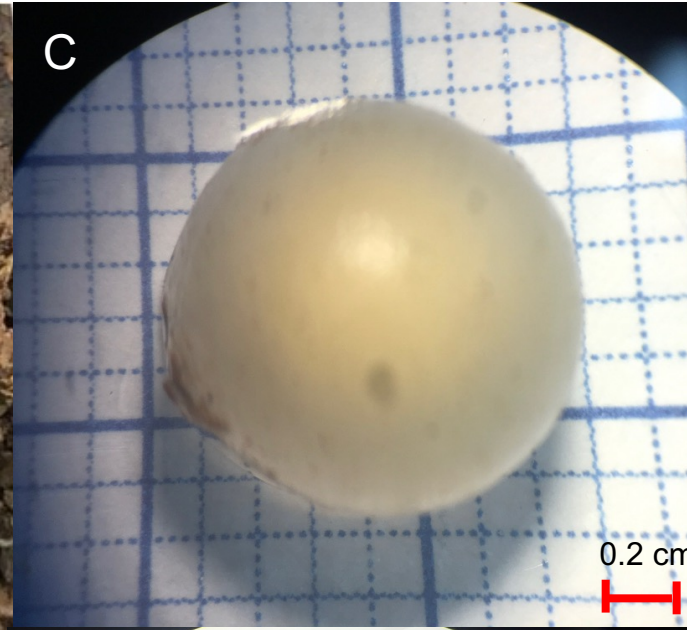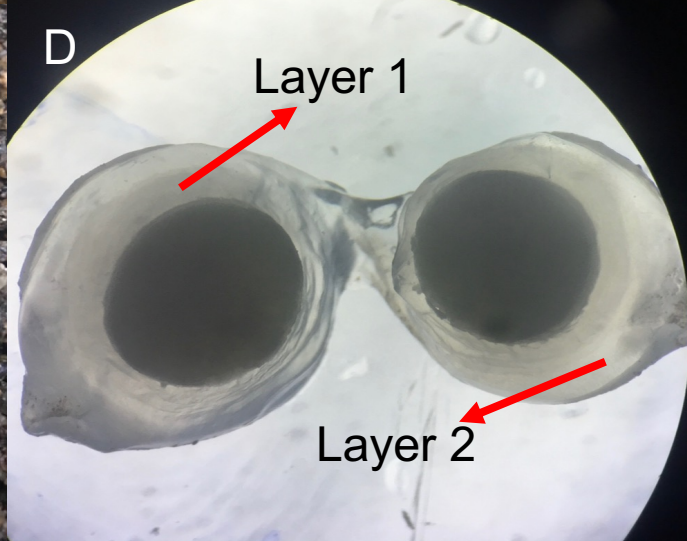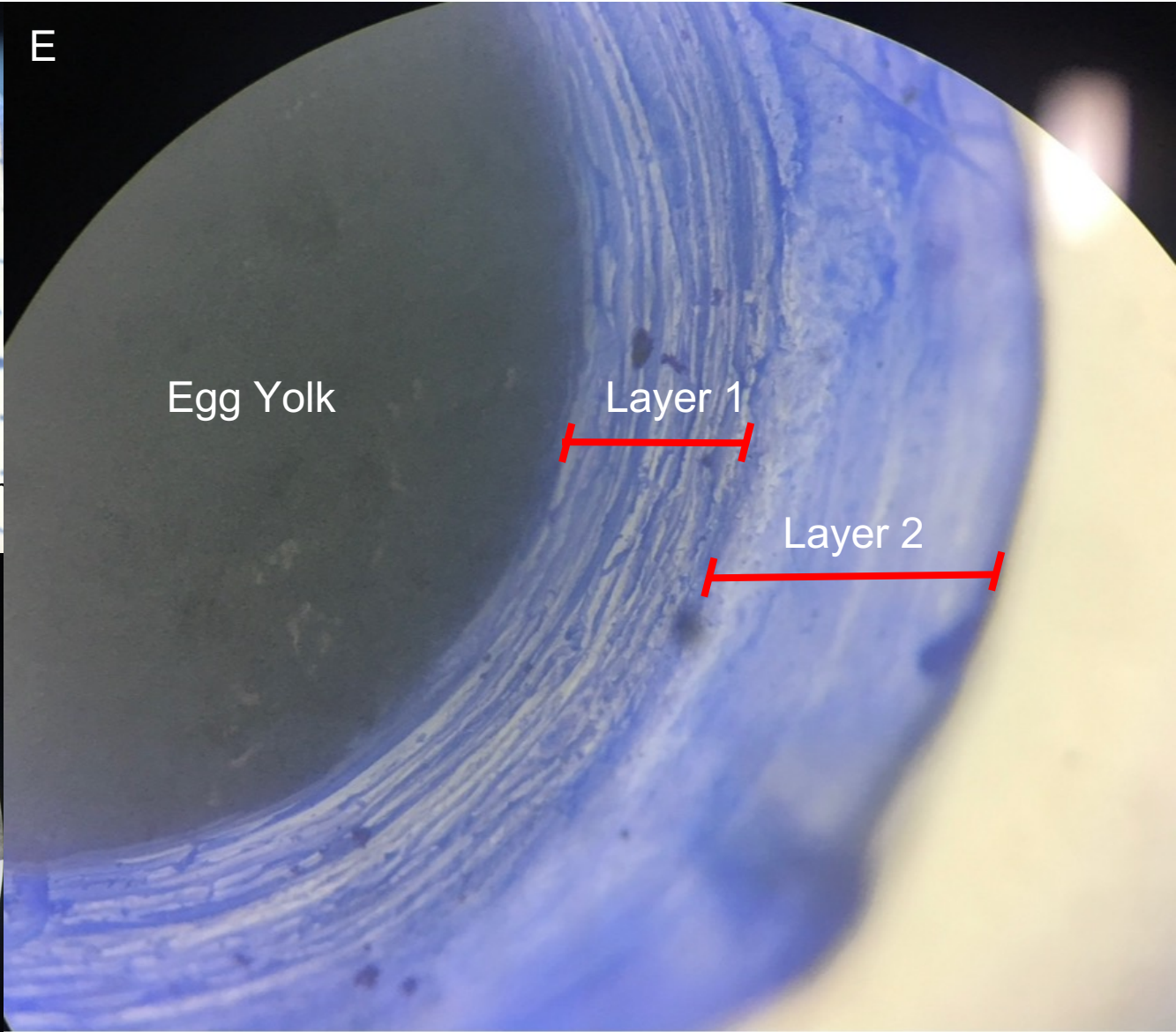

Supplement: Supplementary file 1 [file animals-13-02689-s001.zip › animals-2509587-supplementary.pdf]
